# Transcriptional Responses of *In Vivo* Praziquantel Exposure in Schistosomes Identifies a Functional Role for Calcium Signalling Pathway Member CamKII

**DOI:** 10.1371/journal.ppat.1003254

**Published:** 2013-03-28

**Authors:** Hong You, Donald P. McManus, Wei Hu, Michael J. Smout, Paul J. Brindley, Geoffrey N. Gobert

**Affiliations:** 1 Division of Infectious Diseases & Immunology, Queensland Institute of Medical Research, Brisbane, Queensland, Australia; 2 Department of Microbiology and Microbial Engineering, School of Life Science, Fudan University, Shanghai, China; 3 Queensland Tropical Health Alliance and Centre for Biodiscovery and Molecular Development of Therapeutics at James Cook University, Cairns, Australia; 4 Department of Microbiology, Immunology & Tropical Medicine, School of Medicine & Health Sciences, and Research Center for Neglected Diseases of Poverty, George Washington University, Washington, District of Columbia, United States of America; University of New Mexico, United States of America

## Abstract

Treatment for clinical schistosomiasis has relied centrally on the broad spectrum anthelmintic praziquantel; however, there is limited information on its mode of action or the molecular response of the parasite. This paper presents a transcriptional and functional approach to defining the molecular responses of schistosomes to praziquantel. Differential gene expression in *Schistosoma japonicum* was investigated by transcriptome-wide microarray analysis of adult worms perfused from infected mice after 0.5 to 24 hours after oral administration of sub-lethal doses of praziquantel. Genes up-regulated initially in male parasites were associated with “Tegument/Muscle Repair” and “Lipid/Ion Regulation” functions and were followed by “Drug Resistance” and “Ion Regulation” associated genes. Prominent responses induced in female worms included up-regulation of “Ca^2+^ Regulation” and “Drug Resistance” genes and later by transcripts of “Detoxification” and “Pathogen Defense” mechanisms. A subset of highly over-expressed genes, with putative drug resistance/detoxification roles or Ca^2+^-dependant/modulatory functions, were validated by qPCR. The leading candidate among these was CamKII, a putative calcium/calmodulin-dependent protein kinase type II delta chain. RNA interference was employed to knockdown CamKII in *S. japonicum* to determine the role of CamKII in the response to praziquantel. After partial-knockdown, schistosomes were analysed using IC_50_ concentrations (50% worm motility) and quantitative monitoring of parasite movement. When CamKII transcription was reduced by 50–69% in *S. japonicum*, the subsequent effect of an IC_50_ dosage of praziquantel was exacerbated, reducing motility from 47% to 27% in female worms and from 61% to 23% in males. These observations indicated that CamKII mitigates the effects of praziquantel, probably through stabilising Ca^2+^ fluxes within parasite muscles and tegument. Together, these studies comprehensively charted transcriptional changes upon exposure to praziquantel and, notably, identified CamKII as potentially central to the, as yet undefined, mode of action of praziquantel.

## Author Summary

Schistosome infected mice were treated orally with a single sub-lethal dose of the anthelmintic praziquantel. Parasites were subsequently isolated 0.5–24 hours later and analysed by gene expression microarray. The transcriptional pattern was influenced by the sex of the parasite. Males up-regulated genes associated with surface integrity, muscle function, ion regulation and drug resistance. While for female worms, genes associated with calcium regulation, drug resistance, detoxification and pathogen defense were transcriptionally elevated. Based on these findings, genes with putative ion homeostasis or drug metabolism roles were further investigated for their prospective roles praziquantel action, using the gene silencing procedure RNA interference. The leading gene was CamKII (calcium/calmodulin-dependent protein kinase type II), a member of the Calcium Signalling Pathway. A quantitative assay for parasite motility during praziquantel exposure was used to study worms in which the CamKII gene had been partially silenced. When CamKII transcription was reduced, the effects of praziquantel were augmented, visualised by reduced parasite movement. These functional studies indicate a key role for the kinase CamKII in the mode of action of the praziquantel, and suggest that future studies on this enzyme and other calcium signaling pathway components will be constructive in understanding how praziquantel kills schistosomes.

## Introduction

At least 200 million people are afflicted by schistosomiasis [1], where clinical symptoms associated with the disease range from fever, headache and lethargy, to severe fibro-obstructive pathological changes, portal hypertension, ascites and hepatosplenomegaly, with complications that are frequently fatal. Meta-analysis indicates that the disease burden, in terms of morbidity and mortality is far greater than previously estimated [1]. Current public health approaches to control of schistosomiasis is underpinned by mass or targeted drug treatment with the heterocyclic pyrazino-isoquinoline compound, praziquantel (PZQ). Since the 1980s, treatment for schistosomiasis has relied almost exclusively on this broad spectrum anthelmintic, which is safe, effective against all species, is administered orally, has minimal side effects and is inexpensive. Remarkably, however, there is only limited information on the mode of action of PZQ or how schistosome parasites respond to the drug. With the increasing spread of schistosomiasis and the concomitant extensive deployment of PZQ, a menacing spectre of appearance and spread of drug resistant schistosomes is a worrisome concern.

Early effects of PZQ on *Schistosoma mansoni* worms include contraction and paralysis, which may result from membrane depolarisation and the influx of extracellular calcium [2]. These effects are compounded by uncontrolled muscle tension which results in adult worms being flushed from the mesenteric venules back to the liver, where vacuolisation and disintegration of the schistosome surface and leukocyte migration through the tegument can be readily observed [2]. Sex specific sensitivities for *in vitro* and *in vivo* PZQ exposure are seen in *S. mansoni*; males are more sensitive than females to PZQ [3]. PZQ also disrupts Ca^2+^ homeostasis in schistosomes by an unknown mechanism [4]. Greenberg and colleagues have suggested that PZQ sensitivity in schistosomes is brought about via the beta-subunit variant of the Ca^2+^channel (Ca_v_ß), which results in a massive influx of calcium ions. Competitive binding of Ca^2+^ channels with cytochalasin D (an actin disruptor) interferes with the effects of PZQ, particularly in adult male worms via the disruption of Ca^2+^ homeostasis, subsequently impacting on the tegument actin cytoskeleton [5]. In addition, expression of ATP-binding cassette (ABC) superfamily proteins in schistosomes, including multidrug resistance-associated protein 2 (SMDR2), is altered in worms exposed to sub-lethal levels of PZQ [6], [7]. SMDR2 is also expressed at higher levels in parasite isolates with reduced PZQ sensitivity, and this protein interacts directly with PZQ [6], [7]. Cioli and colleagues [8] hypothesised, however, that calcium influx represents only one component of a complex mechanism which leads to the anti-schistosomal effects of PZQ. Despite these and other documented effects of PZQ, the precise identity and location of the molecular targets of PZQ remain unknown [4].

Genetic crosses of resistant and susceptible strains of *S. mansoni* has revealed that PZQ insensitivity is a quantitative trait, indicating that there may be more than one major physiological target of the drug [9]. Cioli and colleagues [9] speculated that drug metabolism could be the key feature of resistance, rather than the result of significant structural changes to the drug target itself. This notion is supported by earlier findings that revealed an accumulation of fluorescent substrates within the schistosome and an increase in the expression of several ABC transport proteins following exposure to PZQ [10]. Although resistance might also arise from a mutation or structural change in the drug target, resulting in decreased binding [11], other features might be relevant. These could include drug accessibility to tegumental and other schistosome cells. Alternatively, PZQ might be cleared through an up-regulation of antioxidant enzymes. Selective advantage of rare alleles encoding these defences could give rise to multi-drug resistance, as has occurred parasitic nematodes and protozoans [12]. Changes in transcriptional levels of the drug target, rather than a direct mutation, have been suggested as a mechanism for pyrantel resistance in *Ancylostoma caninum* with resistant hookworms down-regulating expression of a nicotinic acetylcholine receptor [13].

Some information is available on transcription of genes associated with calcium homeostasis and putative PZQ resistance mechanisms in *S. mansoni*. One report described transcriptomic responses to PZQ and included *in vitro* culture of adult schistosomes and microarray analysis, which identified 607 up-regulated genes, 247 of which were shown to correlate with known oxidative-stress processes and calcium regulation [14]. PZQ displays a bimodal spectrum of activity, in that it is active against newly transformed schistosomules (<3 days old), inactive against immature 21 day-old worms, and full activity against the sexually mature blood flukes [15]. Recently, Hines-Kay and colleagues utilised transcriptomics to address this refractory/susceptible nature of developmental stages of schistosomes in terms of PZQ activity [16]. The study profiled gene expression in adult and juvenile *S. mansoni* with and without *in vitro* PZQ exposure. The findings suggested that juveniles, which are refractory to PZQ, display enhanced transcriptomic elasticity in the percentage of differentially expressed genes which the authors hypothesise endows the immature stages of schistosomes with the means to withstand the anthelmintic effects of PZQ. Here we describe the use of a novel approach to examine the transcriptional responses of adult *S. japonicum* parasites exposed *in vivo* to a combination of PZQ and the host immune system [17], [18].

## Results

### Microarray analysis

**Figure 1:**
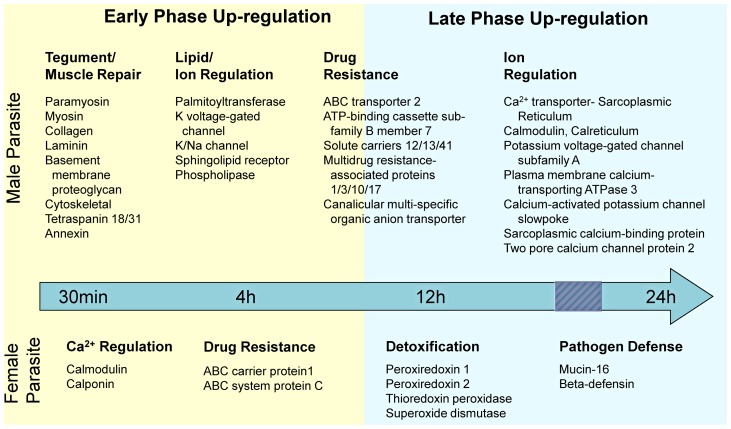
Overview of genes transcriptionally up-regulated in *Schistosoma japonicum* in response to *in vivo* exposure to praziquantel. Broad functions known to be important to schistosome biology, and specific example genes are presented. Responses were noted separately for male and female parasites, and were classified as either “Early Phase” representing time points 30 min and 4 h, or “Late Phase” representative of 12 h and 24 h, after administration of praziquantel. Up-regulation of genes was considered relative to pre-drug administration.

Microarray gene expression analyses were undertaken to investigate the sub-lethal effects of PZQ on *S. japonicum in vivo*. Hybridisations were performed on mRNA isolated from male or female adult parasites for each time point to allow the identification of ±≥2-fold differentially expressed genes, relative to controls (time point 0). For female worms, 264 genes were up-regulated between 30 min and 4 h, increasing to 1,009 genes between 12 and 24 h after drug exposure. PZQ had a broader effect on transcription in males with 1,508 genes up-regulated at 30 min, increasing to 2,718 genes at 24 h post-drug exposure. The number of differentially expressed genes are summarised in Table 1 and notable examples of differentially expressed genes are presented in Table S1. Distinct transcriptional responses by adult *S. japonicum* to PZQ were sex-dependent and varied with the duration of PZQ exposure (Figure 1). A list of all the differentially expressed genes is presented in Table S2. A comparison of similar gene expression patterns between the genders was performed. Using a 2 fold cut of differential regulation compared to time point 0 controls, this included gene expression common to both male and female parasites as grouped for 30 minute or 4 hour time points as early responses and 12 hour or 24 h points as later responses (Table S3). Up-regulated genes (for both sexes) including 19 early, 60 later, and 7 both early and later. By contrast, down-regulated genes common between the sexes included 380 early, 175 later, and 38 that were modulated consistently during early and later time points. Some novel genes were observed both at early and later intervals to be, consistently differentially expressed in both sexes in response to PZQ exposure. This included up-regulated Contig06312 (Dual specificity protein kinase CLK1) which contains a PKc like superfamily motif, and Contig03692 (Early growth response protein 1) which has zinc and nucleic acid binding functions. Down-regulated genes for both sexes and across the entire time course, were more numerous; these included Contig05338 (Exportin-2) a gene related to cell proliferation, and a number of genes associated with ion transport Contig04920 (Cation/acetate symporter) Contig07059 (Solute carrier family 22 member 3) Contig01777 (Probable cation-transporting P-type ATPase) Contig07303 (Uncharacterized symporter) and Contig03785 (Uncharacterized sodium-dependent transporter).

**Table 1:** Numbers of differentially expressed genes in male and female *Schistosoma japonicum* after *in vivo* exposure to praziquantel.

Number of differentially expressed genes (relative to 0 hours).	Time point (hours) and number of differentially expressed genes			
	0.5	4	12	24
Male, Up-Regulated	1519	2450	2295	2735
Female, Up-Regulated	40	233	667	501
Male, Down-Regulated	145	382	1000	862
Female, Down-Regulated	1112	3138	2464	3431

### Calcium Signalling Pathway

**Figure 2:**
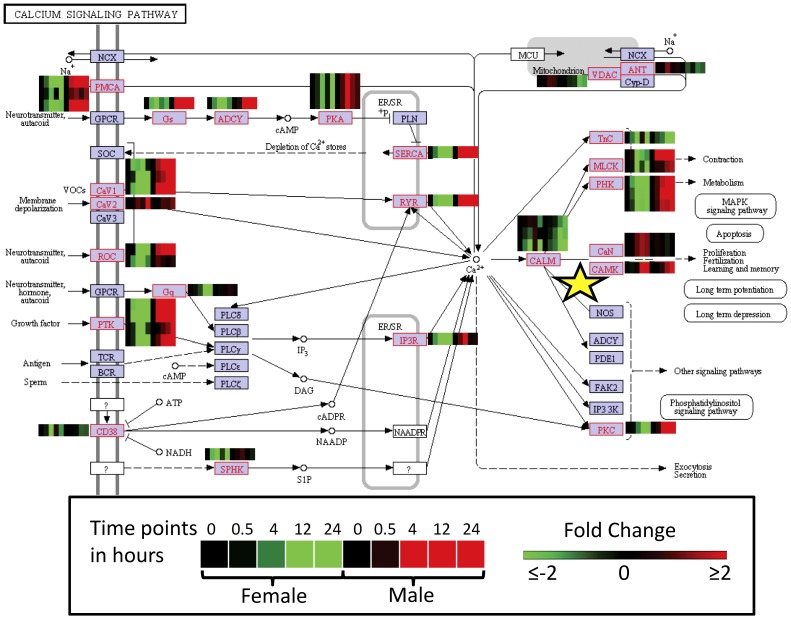
Calcium Signalling Pathway according to KEGG for *S. japonicum* with corresponding heat maps indicating differential gene expression (≥ or ≤2 fold) after *in vivo* exposure of adult male and female parasites with praziquantel for 0, 0.5, 4, 12, 24 h. CamKII is marked with a yellow star.

The differential expression of Contigs with KEGG annotation associated with the “Calcium Signalling Pathway”, were mapped to present an overview of how PZQ impacts on the pathway as a whole, as illustrated in Figure 2. Generally, most genes of the pathway were up-regulated for male *S. japonicum* but down-regulated for female worms over the 24 h observation period, with notable exceptions, including CamKII (Contig01285) which was up-regulated in both males and females (Table S4).

### Real-time PCR validation of microarray findings

To validate these findings of differential expression, nine genes were analysed further by quantitative (q) PCR. The relative fold change of gene expression obtained by microarray and by quantitative PCR was similar for the majority of data points for all nine genes (Figure S1). The microarray and quantitative PCR data sets of the nine genes indicated a significant correlation (alpha = 0.05) between the two methods (Spearman's Rho = 0.83, *P*<0.001, *n* = 90), providing strong support for the integrity of the microarray findings.

### Real time cell assay (RTCA) to assess effectiveness of dsRNA interference and PZQ

Based on microarray data, and then validated by qPCR, we selected five genes that showed a high level of differential expression after exposure to PZQ. The selected candidate genes (Contig02253 *Multidrug resistance protein*, Contigs05840/02748 *ABC transporter H family member 2*) or Ca^2+^-dependant/modulatory functions (Contig10357 *Calretinin*, Contig01285 *Calcium/calmodulin-dependent protein kinase type II delta chain*) (Figure S1) have also been shown to function in either putative drug resistance or detoxification. In general, these genes exhibited increased up-regulation in adult males of *S. japonicum* compared with female worms, a result correlating with our hypothesis that transcriptional responses to PZQ in schistosomes are sex-specific. Subsequently, the impact of these five genes on adult worm survivalibity following drug exposure was evaluated by RNAi, all of which resulted in knockdown, which in the case of CamKII (Ca^2+^/calmodulin-dependent protein kinase II) reached ∼60% (female 69%, 63%, 57%; male 61%, 59% and 50%, in triplicate experiments) when compared with irrelevant (luciferase) controls. The other four candidate genes either did not exhibit marked or as consistent knockdown (not shown) compared to CamKII. Gene silenced worms were examined in motility assays to identify phenotypical differences.

**Figure 3:**
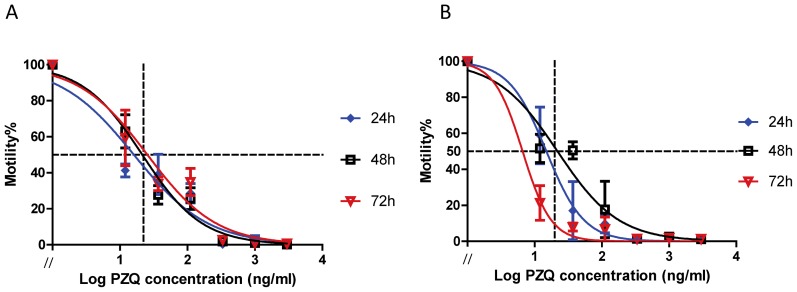
IC_50_ value from real time cell assay of female and male *S. japonicum* treated *in vitro* with praziquantel. Praziquantel dose response curves used to generate IC_50_ values generated from motility index analysis. Panel A: Female worms, Panel B: Male parasite. Error bars (SEM) was shown in the figure every 4 h after been treated with different concentration of praziquantel (0, 12.3, 37, 111, 333, 1000, 3000 ng/ml).

The IC_50_ values for PZQ-treated female and male *S. japonicum* were calculated from motility index analysis using the xCELLigence system (Figure 3). Male and female worms were exposed to 12.3, 37, 111, 333, 1000, 3000 ng/ml PZQ in CSM after which motility was monitored for up to 72 h. After 48 h, 20 ng PZQ/ml reduced movement by ∼50% in both male and female worms (Figure 3A, B).

**Figure 4:**
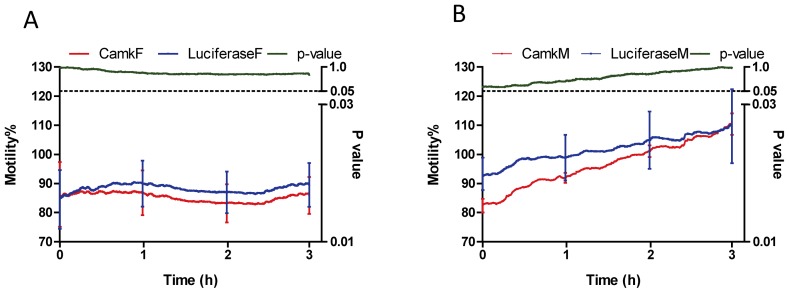
Motility (%) of female and male *S. japonicum* after being electroporated with dsRNA for 48 h–51 h. Motility % is presented on the left y-axis while the corresponding p-value is presented on the right y-axis. Panel A: Female worms, Panel B: Male parasite. Error bars (SEM) was shown at time points, 0, 1, 2 and 3 h.

**Figure 5:**
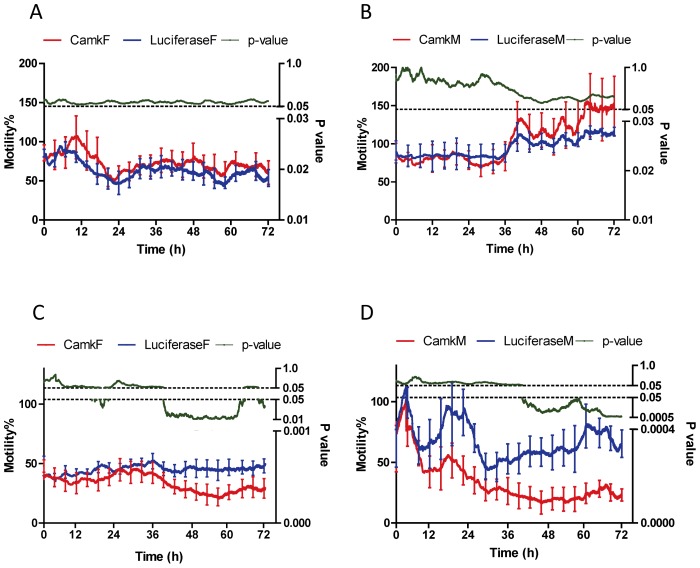
Motility (%) generated by CamKII gene knockdown, female and male worms incubated without or with praziquantel for up to 72 h. Motility % is presented on the left y-axis while the corresponding p-value is presented on the right y-axis. For both sexes, knockdown resulted in no significant changes in mobility without praziquantel treatment. Panel A: Female worms, Panel B: Male parasite without praziquantel treatment. However for both sexes, knockdown leads to less motile worms in the presence of praziquantel. Panel C: Female worms, Panel D: Male parasite with praziquantel treatment. Error bars (SEM) was shown in the figure every 4 h after been treated with or without praziquantel.

To ensure the worms used for PZQ treatment were alive at 48 h after dsRNA electroporation, motility was measured by xCELLigence for 3 h before addition of the drug; the range (% relative to un-electroporated parasites) of motility was for females 86–89% and 85–110% for males (Figure 4). No differences were apparent (*p*-value>0.05) among motility of CamKII and luciferase knockdown groups for both females and males, before addition of PZQ. Only living worms were retained for a further culture in the presence or absence of PZQ and subsequent calculation of the average motility index. Figure 5 shows the changes in the motility of adult males and females with about 60% CamKII knockdown in controls (no PZQ, Figure 5A, B) and with the addition of IC_50_ concentrations of PZQ (5C, D), over 72 h. Worms exhibited decreased motility immediately upon addition of PZQ and, in males, this was followed by spasmodic contraction during e then five hours (Figure 5C, Figure 5D).

Worm motility in the luciferase control groups was consistently maintained at 50–60% in the presence of PZQ, whereas CamKII knockdowns of both male and female parasites displayed further reduction in motility after incubation with IC_50_ PZQ (Figure 5). With the addition of IC_50_ PZQ over 72 h (Figure 5C, Figure 5D), the motility of male worms with CamKII knockdown, relative to luciferase controls, decreased significantly from 61% to 23%; in female parasites motility was reduced from 47% to 27%. Statistical differences of paired treatments at each time point (*p*-value≤0.05) for both male and female parasites, between knockdown groups, was apparent from ∼40 h post PZQ administration, and generally continued until the conclusion of the assay. These results indicated that female and male *S. japonicum* worms, with reduced CamKII levels, were sensitive to IC_50_ PZQ treatment in terms of motility, and both sexes demonstrated a CamKII-dependence in mitigating the effects of PZQ.

## Discussion

This study reports transcriptional and functional approaches to defining the molecular responses of schistosomes to PZQ. We demonstrate the functionally utility of the xCELLigence system to provide real time assessment of motility, a key phenotype of adult *S. japonicum* after PZQ treatment. The findings suggested that specific inhibitors of CamK may increase efficacy of PZQ and that a focus on prospective drug targets in the Calcium Signalling Pathway could facilitate development of improved or alternative anti-schistosomals.

Exposure to anti-parasitic compounds can result in diverse outcomes in terms of the modulation of gene expression. For example, with *Plasmodium falciparum*, chloroquine induces relatively few transcriptional changes (∼100 genes) [19], whereas exposure to artesunate results in ∼400 regulated gene alterations [20]. A microarray-based study of *Trypanosoma brucei* [21] revealed that exposure to thiazolidinediones, and the resultant cellular differentiation, could be attributed to up-regulation of the expression site associated gene 8 (ESAG8). In *S. mansoni*, genes such as glutathione *S*-transferase, are up-regulated in response to xenobiotics [22].

Adult worms of *S. japonicum* respond immediately to exposure to PZQ. A notable feature is rapid disruption of the tegument to expose surface antigens, which has also been linked to perturbation of calcium ion homeostasis [23]. The worms contract, a feature clearly visible to the naked eye, and surface blebbing and other damage is evident by light or electron microscopy [24]–[26]. The concentration of PZQ examined in our study (20 µg/ml) was ∼1000 fold less than that described by Xiao and colleagues [27] who examined tegumental damage in *S. japonicum* adults exposed *in vitro* to the much higher concentrations of 10–30 µg/ml. Xiao and colleagues [27] were able to detect the formation of surface blebs in worms using light microscopy; by contrast, this damage was not apparent in any of the worms examined here (not shown) and we conclude that tegumental damage by PZQ did not occur in control or CamK knockdown parasites.

Our approach to understanding the mechanism of action of PZQ, follows that of others who have investigated the responses of yeast and other microbes in which immediate transcriptional changes occur, reflecting the mode of action of antimicrobial agents [28]. The combination of chemotherapeutic and host immune effects on the schistosomes [17], [18] provides an unique insight into the complete action of PZQ. Our *in vivo* model involved the administration of PZQ to schistosome-infected mice followed by transcriptional analysis of the drug-exposed flukes. The gene expression profiles, which have not been reported previously, demonstrate a polarised response between male and female worms (Table 1 and Table S2).

We hypothesise that schistosomes up-regulate genes to compensate for the effect of PZQ. This phenomenon mirrors that of drug-treated human cells which leads to complex responses upon the binding target [29]. It addition it is apparent that known mechanisms occur which produce a positive feedback loop that modulates the expression level of the respective target protein [29]. Key biological functions associated with ensuring schistosome worm integrity were identified in the transcriptome analysis (Figure 1; Table S1). In adult males, up-regulated genes included, but were not limited to, those associated with tegument and muscle function, lipid and ion regulation, and drug resistance. Whereas fewer genes were up-regulated in females, biological functions related to pathogen defence, general detoxification, drug resistance and Ca^2+^ regulation were prominent.

The schistosome tegument provides structural and functional elements for nutrient uptake and physical and immunological protection, processes for which many components have been identified by other methods including proteomic analysis [30], [31]. Tegument-associated genes up-regulated in male *S. japonicum* included annexins (Contigs00538 and 04019), glucose transporters (Contig05129), calpain (Contig05997), tetraspanins (Contig00678 and 04880) and sodium/potassium-transporting ATPase (Contig05103). Also up-regulated were cytoskeletal components, including tubulin (α, β and δ, Contigs 00204, 00027 and 06580), actin (Contig05360); talin (Contig05315) and supervillin (Contig03361), regulatory elements such as microtubule-actin cross-linking factor 1 (Contig06241) and actin cytoskeleton-regulatory complex protein (Contig02825), and muscle components such as paramyosin (Contig07435), myosin (Contig13962) and actin (Contig05360). Components of the basement membrane, another important tegument structure, including laminin (Contig02502), collagen (Contig06926) and a basement membrane-specific heparin sulfate proteoglycan core protein (Contig11129), were up-regulated early in the response to PZQ by male worms, possibly reflecting tegument stress. Ultrastructural analysis of *S. mansoni* worms has shown that damage to the basement membrane is characteristic of schistosomes exposed to sub-optimal dosages of PZQ [32].

Endocytosis across the schistosome tegument, particularly in males, is a major route of nutrient uptake [33]. Dynamin (Contig01607) and myoferlin (Contig07932) are components of endocytosis within both endothelial and muscle cells of mammals [34]. Both genes were up-regulated in male *S. japonicum* in response to PZQ, suggesting increased tegument activity. This notion was also supported by the over expression of clathrin heavy chain 1 (Contig04218) and phosphatidylinositol-binding clathrin assembly protein (Contig07474), both of which are structural components of coated pits [35]. Thus, while a sub-lethal dose of PZQ appeared to place some facets of tegumental function under stress, resulting in an increase in cytoskeletal elements, other specific processes such as active vesicle-mediated transport appeared to increase.

Ion regulation, drug resistance and immunological defences were phenomena where differential gene expression was prominent for both male and female *S. japonicum*. The majority of the genes within the KEGG *Calcium Signalling Pathway* were differentially expressed, generally resulting in an up-regulation in males and down-regulation in females (Figure 2; Table S4). The effect of PZQ on Ca^2+^ homeostasis is well documented [3], [5], and is confirmed by these findings. However, we show also that the impact on related pathways appears to be sex-specific. Our transcriptional analysis of *S. japonicum* expands on the findings of Aragon *et al.* [14] who studied the gene expression of *S. mansoni* exposed *in vitro* to PZQ. In contrast, we used a mouse model to analyse *in vivo* exposure to PZQ in adult *S. japonicum*. In addition, we performed microarray analyses separately on male and female worms, facilitating description of distinct profiles for each gender. Similarities in the findings of the two studies are, however, evident including the up-regulation of calcium-associated genes (such as Contigs 03004, 08226, 09553) in male schistosomes. Additionally, other ion-associated genes, including those for sodium (Contigs 05931, 0513, 03882) and potassium (Contigs 10776, 10915, 05103, 02734), were up-regulated in both schistosome species. The induction of extracellular superoxide dismutase precursors (Contig04124, 00246), shown in worm pairs of *S. mansoni* [14], was only apparent in female *S. japonicum*. In the calcium pathway, many Ca^2+^-mediated events occur when the released Ca^2+^ binds to and activates the regulatory protein calmodulin (Contig10880), which was strongly up-regulated in both female (93-fold increase) and male (11-fold increase) *S. japonicum* at 24 h post-PZQ treatment *in vivo*. In mammals, calmodulin is thought to activate CamKII (Contig01285) by binding calcium ions [36]. From our findings calmodulin appears as an important component of calcium signaling and in response to PZQ strong up-regulation. However, we did not initially select calmodulin for RNAi since it directly interacts with multiple genes (at least 7 genes downstream) within the pathway, and its knockdown may have presented a much more complicated phenotype. We intend to focus on calmodulin, now that we established protocols and have characterised CamK as a basis for future work.

Other differentially up-regulated genes of note included those encoding mucins (Contigs 04112, 08178 and 07699), a family of proteins which, in schistosomes, may play a role in immune evasion and other host-parasite interactions [37]. Mucins have been shown to be expressed only in the intra-molluscan stages of *S. mansoni* [37] and in the egg, miracidium and sporocyst stages of *S. japonicum* [38]. Their up-regulation, in male and female *S. japonicum* after PZQ exposure is the first indication that mucins are utilised by the mammalian stages as well. The anti-microbial peptide β-defensin (Contig07230) is a key host defence peptide in human neutrophils, and a component of innate immunity, and related peptides perform similar roles for other vertebrates and in invertebrates, fungi and flowering [39]. For example, it serves as anti-microbial role in *Caenorhabditis elegans* and *Ascaris suum* [40]. The current findings represent the first report of up-regulation of this gene in adult schistosomes and may reflect a defense response of the tegument to insult. The identification of these two defence responses in schistosomes emphasises the utility of the *in vivo* PZQ assay presented here.

A central feature of the transcriptional changes in *S. japonicum* subjected to PZQ *in vivo* was the up-regulation of ABC transporters, putative detoxification and multidrug resistance genes. The up-regulation of the ATP-dependent efflux pump SMDR2 in PZQ-treated *S. mansoni in vitro* [6], [7] was mirrored in the *S. japonicum* homologue Contig02253 (Multidrug resistance protein 3) which, in male parasites, was up-regulated ∼4-fold by four hours after drug treatment. The role of peroxiredoxins (Contigs01526 and 11579) in schistosomes has been linked to detoxification, specifically in restricting oxidative damage [41]. Oxidative stress in *S. japonicum* described here may result from the indirect action of PZQ or to immune-mediated damage or a combination of the two.

Efficacy of anthelmintic action can be unambiguously quantified by ascertaining worm motility [42], [43]. To ascertain the real time efficacy of PZQ on *S. japonicum*, we employed the xCELLigence approach recently pioneered by Loukas and colleagues to quantitatively characterize effects of anti-worm drugs by determining worm movements in real time [44]. This system measures conductivity indicative of worm surface contact with the gold electrodes on the surface of the culture plate. The sensitivity of xCELLigence allowed detection of subtle changes in motility in real time for numerous individual worms. Since each worm was contained in a single well, producing its own signal representing motility, this delivered statistically robust observations. To determine a sub-lethal concentration with a 50% reduced motility phenotype, PZQ was used *in vitro* to establish the IC_50_ of 20 ng/ml. This IC_50_ is similar to that used in other studies with schistosomes [6], [7]. It has been demonstrated that when schistosomes are exposed to PZQ *in vitro*, they undergo a rapid influx of calcium ions [45] accompanied by intense muscular paralysis in male worms. This expected response was evident using the xCELLigence approach. The extent of Ca^2+^ overload in mammalian cells is partly mediated by the actions of CamKII, which also participates in regulation of muscle contraction in [46]. Isolated muscle cells from *S. mansoni* exhibit Ca^2+^-dependent contractility [47] but the effect that CamKII has on this process, until now, not been determined. Responsive to fluctuations in Ca^2+^, CamKII functionally modulates many ion channels and transporters in mammalian cells [48]. CaMKII, which exhibits amino acid sequence similarity to the CaMKII auto-inhibitory domain [49], phosphorylates the β2a subunit of voltage gated Ca^2+^ channels to facilitate Ca^2+^ channels. CamKII is necessary for Ca^2+^ homeostasis in mammalian cells and it likely has a similar function in schistosomes, a role particularly important after PZQ exposure.

PZQ produces a well-documented effect on intracellular Ca^2+^ levels in adult schistosomes [50]. Contigs 01107 and 01396, both representing potentially *Voltage-dependent calcium channel subunit alpha*, were up-regulated in males but down-regulated in female *S. japonicum* when exposed to PZQ *in vivo*. The increase in intracellular calcium stimulates activity of the calcium-sensitive proteins, CamKII and protein kinase C (PKC) [51], both of which are involved in the calcium pathway and potential drug targets. PKC (Contig07198, Protein kinase C-like 2), which can act by phosphorylation on voltage-gated Ca^2+^ channel subunits [52], [53], was up-regulated in both male and female *S. japonicum* worms in late response to PZQ. CamKII is known to act on both α and β subunits of voltage-gated Ca^2+^ channels, resulting in the modulation of ion entry into cells [49]. The increase in transcription of both of these kinases in *S. japonicum* suggests that these genes act as a response element to increase Ca^2+^ levels, a known event in PZQ action. It is also apparent that both PKC and CamKII can act on calcium channels themselves. It may be that the interaction with CamKII is needed to restrict the effects of PZQ in schistosomes and when CamKII is reduced, as represented here by RNAi, the motility effects of PZQ are exacerbated. Our data also suggest that a combination of PZQ with CamKII inhibitors such as STO-609 [54], may be synergistic for anti-schistosomal efficacy. However, *S. japonicum* CamKII shares similarity with both human and murine homologs (CAB65123.1: 81% identify, 5e-113 evalue, NP_076302.1: 81% identify, 1e-112 evalue, as determined by Basic Local Alignment Search Tool BLASTp [55]) and thus deployment of inhibitors in the clinic would require careful scrutiny.

Our results partially supports findings of a recent comparison of gene transcription in *S. mansoni* worms exposed to PZQ *in vitro* [16]. Cunningham and co-workers exposed worms to PZQ in a similar time course to that presented here, where their 1 h and 20 h exposures [18] are comparable to the present study's 0.5 h and 24 h intervals. In juvenile *S. mansoni*, 1329 genes at 1 h and 3482 at 20 h were differentially up-regulated, as were 208/ND and 1393/1223 at 1 and 20 h in adult male/female worms [18]. In both developmental stages of *S. mansoni* there was greater differential expression at the later time point, a similar outcome to the present findings. Furthermore, comparison between adult *S. mansoni* male and female parasites showed limited overlap between the sexes - only 20% of same genes were regulated in the same direct (up or down) for both sexes. Although numerous genes were identified in both studies, including ABC transporters, multi-drug resistance genes, and calcium signaling pathway members, many were observed only in juvenile *S. mansoni*, in contrast to the current findings where numerous *S. japonicum* genes in adults were differentially expressed in response to PZQ.

RNAi has been used to suppress a number of schistosome genes so as to investigate their function, but many may not be amenable to exogenous RNA interference [56]. This likely relates to developmental and tissue specificity of the genes. Moreover, refractoriness to RNAi may be due to the secondary structure of transcripts, gene dosage and pathway member redundancy. Moreover, as an informative example of the subtlety of RNAi analysis in schistosomes, whereas suppression of the TGF-β homologue SmInAct lead to a modest 40% suppression at the RNA level, eggs produced by SmInAct knockdown females failed to develop [57]. Accordingly, for other targets in addition to CamKII, we plan in future studies to examine alternative approaches including shRNAi [58], [59].

The data presented here provide new insights into the mechanism of action of PZQ, and the response of schistosomes to the drug. Future investigation will focus on elucidation of the roles of those genes, either as the direct target of PZQ or as member of pathways that are affected by the binding of the drug to its targets. Understanding the emergence of drug resistance in schistosomes requires characterisation of the mode of drug action. If resistance is associated with a mutation of the target(s), identification of other targets within the pathway, demonstrated as critical to parasite survival, would be informative for development of next generation anthelmintics. We re-emphasise that PZQ is the only drug effective against all schistosome species. Should drug-resistance develop, the public health implications would be considerable.

## Materials and Methods

### Ethics statement

The conducts and procedures involving animal experiments were approved by the Animal Ethics Committee of the Queensland Institute of Medical Research (project number A0108-054). This study was performed in accordance with the recommendations in the Guide for the Care and Use of Laboratory Animals of the National Institutes of Health.

### Collection of PZQ-treated worms *in vivo* (sub-lethal effects)

Forty BALB/c mice (female, 6 weeks old) were infected with 30 *S. japonicum* (Anhui, China isolate) cercariae. Six weeks later, 20 of the mice were given a single oral dose of PZQ (300 mg/kg in PBS); the other 20 received PBS. Mice (five per group) were euthanised at four time points (30 min, 4 h, 12 h and 24 h after administration of PZQ or PBS and adult worms recovered by portal perfusion using RPMI 1640 medium at 37°C [38], [60]. The half-life of PZQ in mice is 1–1.5 h [61], and visual effects on the parasite have been reported after 15 min, post subcutaneous administration [32]. Male and female worms were washed separately in 37°C RPMI 1640, pooled for each time point, and stored at ^−^80°C prior to RNA extraction. All parasites were motile at time of storage.

### RNA isolation/labelling and microarray analysis

Male and female adult parasites were separated, pooled (8–10 worms per mouse), stored in RNAlater (Ambion) at −20°C before microarray analysis. Total RNA was isolated from the pooled frozen parasites [62]. RNA quality/quantity was determined using the Bioanalyzer RNA Nano LabChip (Agilent Technologies) and NanoDrop (Thermo Scientific). Labelling (CY3 Agilent One-Color Amp Labeling Kit) and hybridisation methods (Agilent One-Color Microarray-Based Gene Expression Analysis Protocol) were carried using optimised procedures [38].

A 4×44 k feature format microarray was constructed using the *S. japonicum* (Anhui, China isolate) transcriptome [63] by Agilent Technologies custom design and manufacturing pipeline. The array comprises 60-mer oligonucleotide probes for 14,171 SjC contiguous target sequences (Contigs) laid out in triplicate, in addition to proprietary positive and negative controls as supplied by the eArray software interface with Agilent's control grid. Details of the microarray design are available at www.ncbi.nlm.nih.gov/geo/ Accession No. GPL9759 and in Table S5 Series Accession No. GSE41149. Applications of the array for studying different aspects of the biology of *S. japonicum* have been described [38], [64]. Experiments were conducted using an Agilent one-colour protocol and scanned on an Agilent microarray scanner at 550 nm.

### Feature extraction, data analysis

Images from the DNA Microarray Scanner were extracted with Feature Extraction (v10.5). Automatic outliner flagging was used and the list filtered on the basis of *p*-value generated. Feature-extracted data were analysed and visualised using GENESPRING (version 11; Agilent Technologies). Microarray data were normalised using a scenario for ‘Agilent FE one-color’ and ProcessedSignal values were determined using Feature Extraction and GeneSpring microarray software, including signal to noise ratio, spot morphology and homogeneity. ProcessedSignal represents the signal after localised background subtraction and includes corrections for surface trends. Features were deemed ‘Absent’ when the processed signal intensity was less than twice the value of the processed signal error value. Features were deemed ‘Marginal’ when the measured intensity was at a saturated value or if there was a substantial amount of variation in the signal intensity within the pixels of a particular feature. Features that were neither absent nor marginal were deemed ‘Present’. Data points were included only if they were present or marginal, and probes or Contigs were retained if at least half of the data points were ‘Present’. Differential probe hybridisation was statistically evaluated as a *p*-value, and a cut-off value of ≤0.05 in at least 4 of 10 conditions used as the confidence level. Samples were normalised to untreated parasites at time point equals 0, and expressed as a relative fold change on a log_2_ scale. Microarray data were analysed using GeneSpring and calculated *p*-values were used to filter data (≤0.05), carefully considering false-positive results. Multiple testing techniques available via GeneSpring were used including the Benjamini and Hochberg False Discovery Rate [65], [66]. KEGG (Kyoto Encyclopedia of Genes and Genomes) metabolic pathways were considered for the microarray data [67] which have been mapped for *S. japonicum* [68].

### Real-time PCR

The expression profiles of a subset of genes identified during the analysis were validated by real-time PCR. Total RNA samples were DNase-treated (Promega, Annandale, Australia) before complementary DNA (cDNA) synthesis [69]. The SuperScriptTM III protocol with p(dT)15 primers was used to synthesise cDNA. Real-time PCR was performed and analysed as described [60]. Primers used are presented in Table S6; each sample was checked for primer dimerisation, contamination or mis-priming through inspection of its dissociation curve. Contig01379 (DNA double-strand break repair rad50 ATPase) was used as a reference gene for quantitative PCR analyses as the microarray analysis showed constitutive levels of expression of this gene at all time points for both male and female worms after exposure to PZQ. Two independent experiments (from cDNA synthesis) were carried out for the validation of selected genes. Data from the microarray and real time PCR analyses were examined to ascertain if they fitted normal distributions using the D'Agostino and Pearson omnibus and the Shapiro-Wilk normality tests. Statistical analyses were conducted using GraphPad Prism V5 or Microsoft Excel.

### RNAi

Further characterisation of gene function was carried out using RNAi, an approach now feasible for schistosomes, in light of recent advances in knocking down schistosome genes [70], [71]. RNAi was used in conjunction with an *in vitro* assay where *S. japonicum* worms were cultured in the presence of PZQ, so as to clarify the role of specific genes associated with drug action or in PZQ resistance mechanisms. BALB/c mice (females, 6 weeks old) were challenged with 30 *S. japonicum* (Anhui, China isolate) cercariae. Six weeks post-infection mice were euthanised and adult worms obtained by portal perfusion using 37°C RPMI 1640 medium. Adult worms were incubated in complete schistosome media (CSM) containing RPMI 1640 medium, supplemented with 20% (v/v) heat-inactivated fetal calf serum, 100 IU/ml penicillin and 100 µg/ml streptomycin, at 37°C in an atmosphere of 5% CO_2_ in air overnight [72]. dsRNAs were transcribed *in vitro* from template PCR products using gene-specific primers tailed with the T7 promoter sequence. Briefly, luciferase dsRNA (dsLUC) was used as a negative control, as reported in other studies with schistosomes [70], [71]. dsRNA of CamKII (contig01285) was synthesised from *S. japonicum* cDNA using gene-targeted primers containing T7 promoter sequences:

(F: 5′-TAATACGACTCACTATAGGGAGAGAAGATGGCTACTTCTGTACTCC-3′;

R: 5′-TAATACGACTCACTATAGGGAGATTCCATACGGTTCTTTGCGTAAAA-3′).

dsRNA was synthesised and purified using a Megascript RNAi kit (Ambion). Five pairs of worms in 50 µl electroporation buffer [73], containing 0.5 µg/µl long dsRNA, were electroporated in a 4 mm cuvette by applying a square wave with a single 20 ms impulse at 125 v [74]. Following electroporation, parasites were transferred to 150 µl pre-warmed (37°C) CSM. After overnight culture, media were replaced with 300 µl of CSM. Worms were collected at 48 h post-treatment with dsRNA, and male and female worms were separated for total RNA extraction. Gene transcript levels were measured by real time PCR, with NADH- ubiquinone reductase included as the reference gene [38].

### Motility assay for schistosomes

Adult worms were perfused from mice with 37°C RPMI, as described above for RNAi. The motility of adult male or female *S. japonicum* and IC_50_ values were assessed using the xCELLigence system (Roche Inc.) [44]. RTCA controller software (Roche Inc.) was used to determine how the information was gathered from the 96 well E-plate (Roche Inc.). For real-time monitoring of parasite motility, individual female or male worms were cultured *in vitro* in a 96 well E-plate (one worm per well). Each worm was cultured in 180 µl of CSM per well and motility was monitored every 15 seconds for 3 h to obtain a baseline motility (to identify healthy parasites) reading prior to the addition of 20 µl of a 10× solution of PZQ (stock solution in 100% ethanol at 5 mg/ml). Before the motility of multiple parasites in a treatment group was combined to produce an average and standard deviation (SD), we manually curate the data to identify any worms that had died or was severely damaged by handling either at the baseline collection or during the subsequent culture. As such the data from that specific e-plate well (representing the dead parasite) were removed from further analysis. The motility index of each worm was calculated, for the 3 h prior to the addition of PZQ, as the SD over 150 data points of the cell index (CI) difference from the rolling average (average of the 10 proceeding and preceding CI values - 5 min total) over 20 data points [44]. For generation of the IC_50_ of PZQ *in vitro*, a final working concentration range of 12.3–3,000 ng/ml PZQ was used (Figure S2). After addition of PZQ, worms were monitored every 15 seconds for a further 72 h; motility index was calculated as the SD over 800 data points, and the CI difference from the rolling average over 20 data points. We were able to use 800 data points, and thus more accurately determine SD, due to the longer time course of 72 h. Dead worms (heat killed) were included as immobile controls and considered to exhibit 0% motility. Positive control worms (without PZQ) were cultured in the presence of the ethanol concentration equivalent to that for the highest drug concentration, and represented 100% worm motility. A log_10_ (drug concentration) versus normalised response (100%-0%) formula with variable slope and automatic removal of outliers. Statistical analyses were undertaken using Graphprism 5.0 [44]. The Hill Slope and LogIC_50_ value were used together and compared for significant differences using an extra sum-of squares *F*-test.

For RNA interference, worms were cultured for 48 h after dsRNA treatment; individual female or male worms were transferred to the E-plate in 180 µl medium per well to monitor mobility, as above. Briefly, worms were monitored for 3 h to obtain a baseline motility reading before addition of PZQ (IC_50_ concentration of 20 ng/ml). After adding the drug, worms were monitored for 72 h. Worms subjected to dsRNA but not PZQ served as knockdown controls, and allowed the differentiation of the separate effects of PZQ incubation and RNAi on motility. The motility index and motility (%) of treated or untreated worms were determined as described. For each assay, 6–10 worms were monitored simultaneously and separately for each sex and treatment group (with or without PZQ). A *t*-test (two-tailed, two-sample equal variance) for each time point was undertaken (in Microsoft Excel) to evaluate significance of differences in motility between treatment groups.

## Supporting Information

Figure S1 qPCR validation of a subset of genes identified from the microarray analysis. qPCR results are presented as vertical bars, while the microarray results are presented as a continuous line. All data are normalised to time point 0, separately for female and male parasites.
(TIF)

Figure S2 Motility index of female and male *Schistosoma japonicum* treated without or with different concentration (series dilution from 12.3 ng/ml to 3000 ng/ml) of praziquantel for 72 h. Panel A: Female worms, Panel B: Male parasite. Error bars (SEM) was shown in the figure every 4 h after been treated with or without praziquantel.
(TIF)

Table S1 Novel genes associated with praziquantel exposure of adult *Schistosoma japonicum in vivo*. Early (0.5 h to 4 h) and later (12 h to 24 h) responses for female and male parasites are presented normalised to time point 0 (expression = 1) for each sex as a fold ratio.
(XLS)

Table S2 All 5,097 genes that passed flag and p-value≤0.05 filtering in 10 of 20 samples. Normalised data to time point 0 for adult female and male parasites and corresponding t-test p-value and flag result for each time point. Description of Contigs are also included. P = present, A = absent, M = marginal. Microarray Contig number and gene expression fold change relative for time point 0 for male (M) and female (F) parasites at time points 0, 0.5, 4, 12 and 24 h.
(XLSX)

Table S3 Gene expression details of early (0.5 or 4 h) and later (12 or 24 h) differentially expressed gene after praziquantel exposure, for both adult male and female *S. japonicum* parasites. A 2 (or 0.5) fold cut off was applied. The corresponding common gene description, microarray Contig number and gene expression fold change relative for time point 0 for male (M) and female (F) parasites at time points 0.5, 4, 12 and 24 h.
(XLSX)

Table S4 Gene expression details for the Calcium Signalling Pathway (presented in Figure 2), after praziquantel exposure, for adult male and female *S. japonicum* worms. The corresponding KEGG number, common gene name, microarray Contig number and gene expression fold change relative for time point 0 for male (M) and female (F) parasites at time point 0, 0.5, 4, 12 and 24 h are presented.
(XLSX)

Table S5 Microarray Design file. Description: OS = Organism Nam, GN = Gene Name, PE = Protein Existence, SV = Sequence Version. Systematic: Probe identifiers. Protein Identifier: BLASTX annotation result based on protein sequence. Synonym: probe identity. Microarray Description: BLASTn annotation result based on nucleotide sequence.
(XLSX)

Table S6 Primers used for qPCR validation.
(XLSX)
