# Supplementary figures and images for: Transcriptional Responses of In Vivo Praziquantel Exposure in Schistosomes Identifies a Functional Role for Calcium Signalling Pathway Member CamKII
Source: PLoS Pathog. 2013 Mar 28;9(3):e1003254. doi: 10.1371/journal.ppat.1003254 (PMC3610926; doi:10.1371/journal.ppat.1003254)

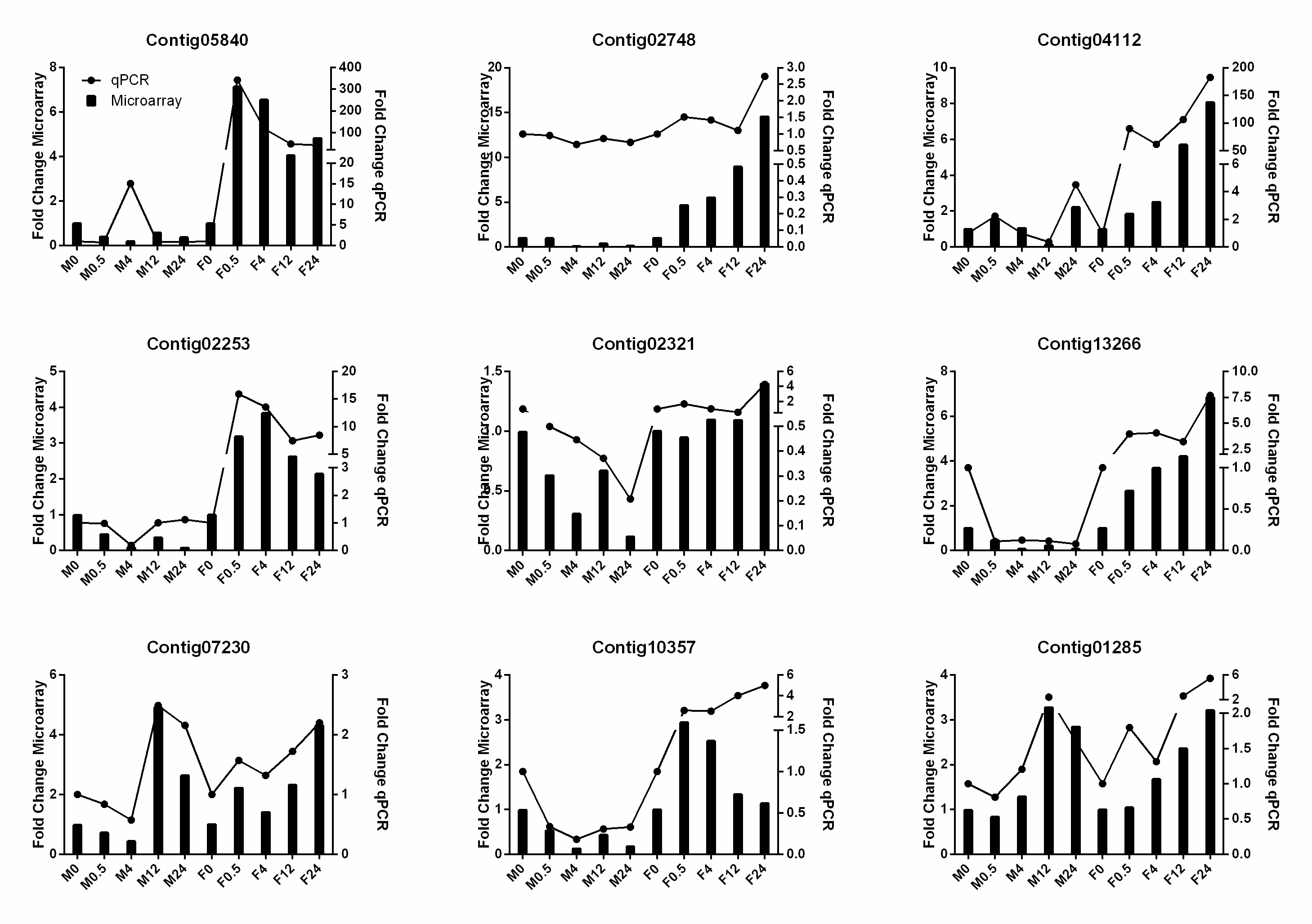

Supplement: Figure S1 — qPCR validation of a subset of genes identified from the microarray analysis. qPCR results are presented as vertical bars, while the microarray results are presented as a continuous line. All data are normalised to time point 0, separately for female and male parasites. (TIF) [file ppat.1003254.s001.tif]

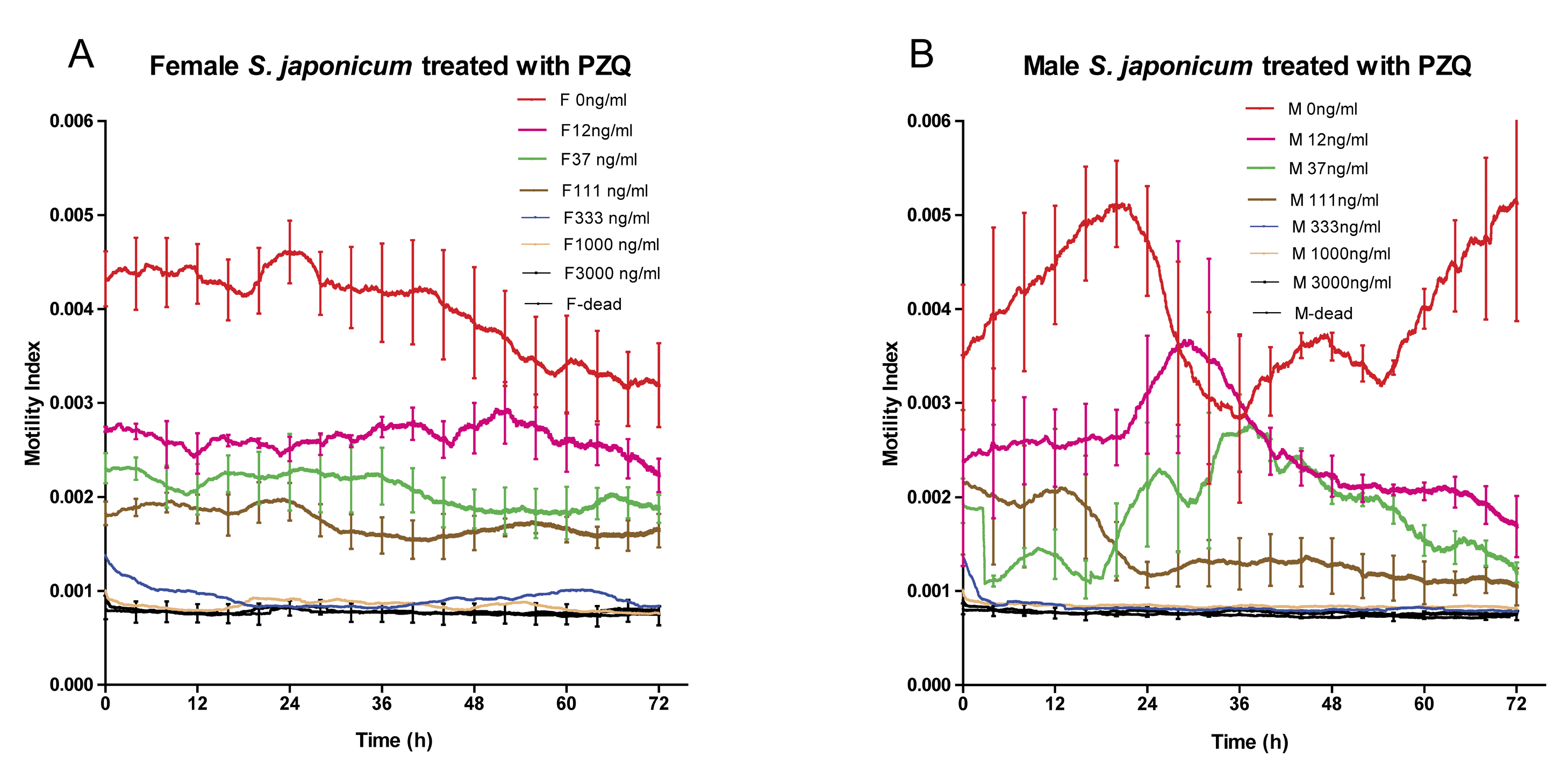

Supplement: Figure S2 — Motility index of female and male Schistosoma japonicum treated without or with different concentration (series dilution from 12.3 ng/ml to 3000 ng/ml) of praziquantel for 72 h. Panel A: Female worms, Panel B: Male parasite. Error bars (SEM) was shown in the figure every 4 h after been treated with or without praziquantel. (TIF) [file ppat.1003254.s002.tif]
